# Metabolomics Insights into Salivary Profile in Dogs with *Babesia canis* Infection

**DOI:** 10.3390/biom15040520

**Published:** 2025-04-01

**Authors:** Josipa Kuleš, Ivana Rubić, Dina Rešetar Maslov, Maša Efendić, Krešimir Martinković, Elizabeta Pongrac, Iva Šmit, Dalibor Potočnjak, Renata Barić Rafaj, Vladimir Mrljak

**Affiliations:** 1 Department of Chemistry and Biochemistry, Faculty of Veterinary Medicine, University of Zagreb, 10 000 Zagreb, Croatia; rrafaj@vef.unizg.hr; 2 Internal Diseases Clinic, Faculty of Veterinary Medicine, University of Zagreb, 10 000 Zagreb, Croatia; irubic@vef.unizg.hr (I.R.); drmaslov@vef.unizg.hr (D.R.M.); mefendic@vef.unizg.hr (M.E.); epongrac@vef.unizg.hr (E.P.); ismit@vef.unizg.hr (I.Š.); dpotocnjak@vef.unizg.hr (D.P.); vmrljak@vef.unizg.hr (V.M.); 3 Department for Microbiology and Infectious Diseases with Clinic, Faculty of Veterinary Medicine, University of Zagreb, 10 000 Zagreb, Croatia; kmartinkovic@vef.unizg.hr

**Keywords:** vector-borne diseases, saliva metabolome, dog

## Abstract

Babesiosis is a significant vector-borne zoonotic disease with major global economic and health implications, affecting various vertebrate hosts. Babesia parasites are auxotrophic for essential nutrients, relying on their hosts for metabolic support. This study investigated salivary metabolomic changes in dogs naturally infected with *Babesia canis* (N = 14) compared to healthy controls (N = 14) using untargeted and targeted mass spectrometry-based approaches. Saliva, a biofluid rich in metabolites, undergoes alterations in response to systemic diseases, making it a promising medium for studying host–pathogen interactions. Metabolomic profiling was performed using a Dionex UltiMate 3000 UHPLC system coupled with a Thermo Orbitrap Q Exactive mass spectrometer. An untargeted analysis detected 2257 salivary features, of which, 868 were significantly altered, with seven metabolites identified by reference to standards. A targeted analysis revealed significant changes in seven metabolites. Functional bioinformatics indicated disruptions in amino acid, nucleotide, and lipid metabolism, alongside alterations in energy production pathways, and purine metabolism. These findings provide critical insights into the metabolic shifts underlying canine babesiosis, supporting the development of advanced diagnostic and therapeutic strategies in the future. This study highlights the intricate interplay between host and pathogen, particularly in nutrient acquisition and metabolic regulation.

## 1. Introduction

Apicomplexan parasites represent a significant group of pathogens, known for their widespread impact on global health and morbidity [1]. Among these, the species of the genus *Babesia* are particularly notable for their ability to infect red blood cells (RBCs) in a variety of animal hosts, leading to substantial economic and health challenges worldwide [2]. In Europe, *Babesia canis* is recognized as the primary causative agent of canine babesiosis, with documented infections across multiple countries and prevalence rates varying between 2.3% and 44.8%. The increasing incidence and geographic spread of canine babesiosis are largely driven by expanding tick habitats and increased animal mobility [2].

These parasites are auxotrophic for many essential nutrients, relying heavily on their host for metabolic support [3]. The metabolites necessary for *Babesia* survival may be scavenged directly from the host or synthesized using available precursors. As is characteristic of many host–pathogen interactions, *Babesia* must divert host resources to sustain its growth, leading to metabolic disturbances within hosts that struggle to maintain homeostasis. Measurable effects, such as hemolysis, hemoglobinuria, reduced hematocrit levels, low hemoglobin concentrations, elevated reticulocyte counts, and increased lactate dehydrogenase levels, exemplify the pathological consequences stemming from the metabolic interplay between *Babesia* and its host [4,5].

The metabolome represents the final product of intracellular biochemical processes and is highly responsive to environmental changes, including stress induced by infections. Metabolomic analyses have demonstrated significant value in elucidating the pathological mechanisms underlying infectious parasitic diseases [6]. The application of metabolomics, which involves the comprehensive analysis of metabolite levels, has emerged as a vital tool for investigating host–parasite interactions in apicomplexan infections. This approach holds significant potential for the development of urgently needed therapeutics and improved diagnostic methods. Advances in research over the past decade have facilitated a systems biology perspective on apicomplexan parasites, with metabolomics providing a critical insight into their metabolic activities.

Recent metabolomic research has increasingly focused on deciphering host–parasite interactions. Given that parasites inherently manipulate their host’s metabolism to their advantage, understanding these dynamics is crucial for elucidating disease pathology. Numerous studies have employed nuclear magnetic resonance (NMR) spectroscopy to analyze the systemic effects of parasitic infections in vivo, revealing significant metabolic modulation by various parasites, such as *Schistosoma*, *Trichinella*, and *Plasmodium* [7,8,9]. These investigations have highlighted metabolic changes that traditional biochemical methods might overlook.

The genomes of various *Babesia* species, including *B. canis*, are among the smallest recorded within apicomplexans, reflecting their minimal metabolic pathways and auxotrophic lifestyles, which are reliant on host-derived nutrients [3,10]. Infection with *B. divergens*, which infects human erythrocytes, leads to significant changes in the host metabolome, particularly with respect to lipids and enery flux pathways [3]. Acute *B. microti* infection significantly impacted the mouse serum metabolome, leading to disturbances in taurine and hypotaurine metabolism, histidine metabolism, and arachidonic acid metabolism [11].

Recent studies utilizing metabolomics have demonstrated that *B. canis* infection leads to significant alterations in the serum and urine metabolome of infected dogs. For instance, a study involving 12 dogs naturally infected with *B. canis* identified a total of 295 metabolites using various analytical platforms, including ultra-high performance liquid chromatography–tandem mass spectrometry (UHPLC-MS/MS) and gas chromatography–mass spectrometry (GC-MS) [12]. These metabolites were indicative of the disrupted metabolic pathways associated with the infection, such as glutathione metabolism, amino acid metabolism, and energy production pathways. In dogs with babesiosis and different degrees of kidney function, untargeted and targeted MS-based metabolomics approaches revealed changes in the urinary metabolome associated with the inflammatory host response, oxidative stress, and energy metabolism modulation [13]

The development of diagnostic methodologies that are timely, cost-effective, accurate, and noninvasive remains a critical focus for clinicians and researchers. Saliva, as a biological fluid, holds significant potential due to its diverse array of metabolites, which are known to undergo alterations in response to both local and systemic diseases [14]. The salivary glands are highly vascularized, facilitating the exchange of blood-derived constituents. It is hypothesized that molecules from the bloodstream enter salivary tissues through transcellular mechanisms or paracellular pathways (e.g., extracellular ultrafiltration), thereby influencing the molecular composition of saliva [15,16,17]. This molecular content is thought to reflect an individual’s physiological and pathological state. Such insights have established the foundation of salivary diagnostics, leading to extensive research into saliva-based biomarkers for a wide range of conditions, including cancer and infectious diseases [14]. The use of saliva as a diagnostic fluid offers several advantages over serum or plasma in clinical studies. Saliva collection is noninvasive, straightforward, and does not require trained healthcare personnel, thereby minimizing patient discomfort and facilitating sample collection in various settings. Additionally, saliva sampling is cost-effective, with lower collection and storage costs compared to blood samples, making it a practical option for at-home monitoring or in-clinic use without the need for specialized equipment. Overall, saliva presents a promising alternative to plasma for disease detection and monitoring, offering a more accessible, economical, and patient-friendly approach, as reported in recent saliva metabolomics studies in dogs [18,19,20,21,22].

Building on these insights, the present study aimed to investigate alterations in the salivary metabolome of dogs naturally infected with *B. canis* compared to healthy controls. This was achieved using both untargeted and targeted mass spectrometry-based metabolomics approaches. A functional bioinformatic analysis of identified metabolites and the associated pathways provided critical insights into the mechanisms underlying host–parasite interactions and the pathogenesis of babesiosis.

## 2. Materials and Methods

### 2.1. Animals and Sample Collection

The dogs in this study were categorized into two groups: a control group and a *B. canis*-infected group. The control dogs were examined at the Internal Diseases Clinic as part of routine health assessments, while the *B. canis*-infected dogs were admitted for clinical treatment. The study protocol was approved by the Committee on the Ethics of the University of Zagreb, Faculty of Veterinary Medicine (Permit Number: 640-01/18-17/63, 19 September 2018).

Babesiosis was diagnosed based on the identification of parasites within infected erythrocytes on thin blood smears, which were stained using May–Grünwald–Giemsa stain (Merck, Darmstadt, Germany). Further confirmation and subspecies identification were performed with polymerase chain reaction (PCR) analysis, as described in previous studies [23]. The study included 14 dogs of various breeds with naturally occurring babesiosis caused by *B. canis*, comprising nine females and five males, with a median age of 4.5 years (range: 1–12 years). According to the diagnosis, all the infected dogs were administered a single subcutaneous dose of imidocarb dipropionate (Imizol^®^ 12%, Schering-Plough, Kenilworth, NJ, USA) at a dosage of 6 mg/kg on the day of admission.

The control group comprised 14 healthy dogs, age- and sex-matched to the babesiosis group. Routine hematological and biochemical analyses were performed to assess the health status of the animals. The control group was tested for the presence of various parasitic infections and had no history of illness in the past three months.

Saliva samples were collected from all the dogs by placing a small sponge in their mouth until it was fully moist. None of the dogs in this study showed clinical signs of periodontitis. The sponges were then placed in Salivette collection devices (Salivette, Sarstedt, Aktiengesellschaft & Co., Nümbrecht, Germany) and kept on ice until centrifugation at at 3000× *g* for 20 min at 4 °C. After centrifugation, the saliva samples were transferred to Eppendorf tubes and stored at −80 °C until metabolomic analysis. Simultaneously, a blood sample was taken from each dog for the biochemical analysis to rule out any organ-related diseases. All the dogs underwent an overnight fast before the collection of both the blood and saliva samples.

### 2.2. Untargeted Metabolomics Analysis

#### 2.2.1. Sample Preparation and LC-MS/MS Analysis

Saliva samples for the untargeted metabolomics analysis were prepared according to a previously published protocol [13]. For metabolite extraction, a solvent mixture of chloroform, methanol, and water (1:3:1, *v*/*v*/*v*) was utilized. The chloroform and methanol were obtained from Honeywell (Charlotte, NC, USA), and the water was sourced from Merck (Darmstadt, Germany). Briefly, 1000 µL of ice-cold extraction solvent was added to 25 µL of each saliva sample, followed by vortexing on a cooled mixer at 4 °C for 5 min. Additionally, a pooled quality control sample was prepared by combining 10 µL from each individual sample, from which 25 µL was subjected to the same extraction procedure. All the samples (individual, pooled, quality control, and matrix blank) were subsequently vortexed and centrifuged at 13,000× *g* for 5 min at 4 °C, and the supernatant containing the extracted metabolites was analyzed.

The metabolite analysis was conducted using a Dionex UltiMate 3000 UHPLC system (Thermo Fisher Scientific, Germering, Germany) coupled with a Thermo Orbitrap Q Exactive mass spectrometer (Thermo Fisher Scientific, Bremen, Germany). Metabolite separation was achieved through hydrophilic interaction liquid chromatography (HILIC) on a ZIC-pHILIC column (150 mm × 4.6 mm, 5 µm; Merck Sequant, Darmstadt, Germany). Mobile phase A consisted of 20 mM ammonium carbonate in water, and mobile phase B was 100% acetonitrile. The metabolites were eluted with a linear gradient from 80% to 5% of phase B at a flow rate of 0.3 mL/min.

The Orbitrap Q Exactive mass spectrometer operated in both positive and negative ionization modes with a mass resolution of 70,000, covering a mass-to-charge ratio (*m*/*z*) scan range of 70–1050. The source voltage was set to +3.8 kV in positive mode and −3.8 kV in negative mode. Additional parameters included a sheath gas flow rate of 40 arbitrary units, an auxiliary gas flow rate of 5 arbitrary units, and a capillary temperature of 320 °C. A standard mix of 148 reference compounds for metabolite identification was provided by Glasgow Polyomics (Glasgow, UK) and analyzed alongside the samples.

#### 2.2.2. Data Processing for Untargeted Metabolomics

Metabolomics data pre-processing, including alignment, batch correction, and identification, was conducted using the Polyomics Integrated Metabolomics Pipeline (PiMP), accessible at http://polyomics.mvls.gla.ac.uk (accessed on 7 June 2024). This R-based pipeline utilizes XCMS for feature detection and mzMatch.R for standard metabolomics data pre-processing tasks [24]. The metabolite identification was performed by matching the retention times/masses or masses of detected peaks to reference standards, while feature annotation was achieved using the Human Metabolome Database (HMDB) and/or the Kyoto Encyclopedia of Genes and Genomes (KEGG).

### 2.3. Targeted Metabolomics Analysis

#### 2.3.1. Sample Preparation and Mass Spectroscopy Analysis (FIA-MS/MS and LC-MS/MS)

Sample preparation was carried out using the AbsoluteIDQ p400 HR kit (Biocrates Life Sciences AG, Innsbruck, Austria) for targeted metabolomics analyses of up to 408 metabolites across 11 classes, following the manufacturer’s protocol. This kit enabled the detection and quantification of various metabolite classes via combined liquid chromatography–mass spectrometry (LC-MS/MS) and flow-injection analysis–mass spectrometry (FIA-MS/MS). LC-MS/MS was used for amino acids and biogenic amines, while FIA-MS/MS quantified acylcarnitines, glycerophospholipids, glycerides, hexoses, cholesteryl esters, and sphingolipids.

Saliva samples (10 µL each), calibration standards, zero standards (phosphate-buffered saline), and quality control samples were added to the 96-well plate, along with a blank sample. The samples were dried under a vacuum for 30 min (Thermo Scientific, Waltham, MA, USA) and then derivatized with 50 µL of 5% phenylisothiocyanate (PITC) (Sigma-Aldrich, St. Louis, MO, USA) in a water/ethanol/pyridine mixture (1:1:1; ethanol from Honeywell, Charlotte, NC, USA; pyridine from BDH PROLABO, Lutterworth, UK). After incubation for 20 min, the samples were dried again under a vacuum for 60 min.

Metabolite extraction was performed by adding 300 µL of 5 mM ammonium acetate (Sigma-Aldrich, St. Louis, MO, USA) to methanol (Honeywell, Charlotte, NC, USA), followed by shaking for 30 min. The extracts were then collected under a vacuum. For FIA-MS/MS, 250 µL of the FIA mobile phase was added to each sample. For the LC-MS/MS analysis, 150 µL of the extract was diluted with 150 µL of LC-MS-grade water.

Metabolite extracts, standards, and QC samples were analyzed using a Dionex Ultimate 3000 UHPLC system (Thermo Fisher Scientific, Germering, Germany) coupled with a Q Exactive Plus mass spectrometer (Thermo Fisher Scientific, Bremen, Germany). Separation was performed on a Thermo p400 HR UHPLC column (Biocrates, Innsbruck, Austria), with the column temperature maintained at 50 °C. Mobile phase A was composed of 0.2% formic acid in water (Merck, Darmstadt, Germany), while mobile phase B contained 0.2% formic acid in acetonitrile (Honeywell, Charlotte, NC, USA). A 5 µL injection volume was used, with a total run time of 5.81 min. The gradient increased from 0 to 95% mobile phase B over 4 min at a flow rate of 0.8 mL/min.

For the FIA-MS/MS analysis, the metabolites were eluted using the FIA mobile phase with an initial flow rate of 0.05 mL/min for 1.6 min, increased to 0.2 mL/min for 1.2 min, and then reduced back to 0.05 mL/min. The analyses were performed in both positive and negative ionization modes for LC-MS/MS and FIA-MS/MS, following the Biocrates’ guidelines (Biocrates Life Science AG, Innsbruck, Austria). The Orbitrap mass spectrometer was operated in full-scan mode with electrospray ionization at a resolution of 70,000. The scan range was set to *m*/*z* 100–800 for LC-MS/MS and *m*/*z* 100–1000 for FIA-MS/MS. Key operational parameters included one microscan, an automatic gain control (AGC) target of 1 × 10^6^, and a maximum injection time of 250 ms.

#### 2.3.2. Data Processing for Targeted Metabolomics

The quantification of the LC-MS metabolites was performed in XCalibur Quan version 4.1 software (Thermo Fisher Scientific, Waltham, MA, USA), employing a seven-point calibration curve and isotope-labeled internal standards for most analytes. The FIA-MS/MS analysis utilized a single-point calibrator with representative internal standards. The data analysis was performed according to the manufacturer’s guidelines using Biocrates MetIDQ software (version Boron, Biocrates Life Science AG, Innsbruck, Austria) for data processing, quality assessment, and export.

### 2.4. Statistical and Bioinformatic Analysis

All the statistical and bioinformatic analyses were conducted using the online software MetaboAnalyst v.6.0 [25].

For the untargeted metabolomics dataset, the analysis was conducted on the combined positive and negative ion datasets, which were exported as a peak intensity table from PiMP. Missing values were replaced by 1/5 of the minimum positive value for each variable. The intensities of the extracted metabolites were log-transformed and Pareto-scaled. The differences in the metabolic profiles between the control and the *B. canis*-infected groups were assessed using a *t*-test (*p* < 0.05). 

For the targeted metabolomics dataset, metabolites with concentrations below the limit of detection (LOD) in more than 50% of the samples within a group, or those for which the blank measurements were outside the acceptable range, were excluded from analysis. Subsequently, missing values were imputed using the KNN method, and the concentrations were log-transformed and Pareto-scaled. Univariate and multivariate statistical approaches were employed for selecting the most significant changes in the metabolite concentrations between the control dogs and the dogs with *B. canis* infection.

A Metabolite Set Enrichment Analysis (MSEA) of the significant metabolites identified by both the untargeted and the targeted approach were examined with the pathway-associated metabolite sets (SMPDB; 99 metabolite sets based on normal human metabolic pathways, set as a metabolite set library) in order to increase the robustness and accuracy of the analysis.

Correlations between the serum creatinine concentrations assessed by a commercial kit on an automated chemistry analyzer (Architect c4000, Abbot, IL, USA) and the salivary concentrations assesed by the targeted metabolomics approach were calculated using the statistical software GraphPad Prism version 5 (GraphPad Software Inc., San Diego, CA, USA).

## 3. Results

### 3.1. Untargeted Metabolomics

The untargeted metabolomics analysis resulted in the detection of 2257 features in the saliva samples (Supplementary Table S1). A total of 35 features were identified using retention time and *m*/*z* (Level 1 MSI) and other features were annotated using *m*/*z* only (Level 3 MSI) (Supplementary Table S2). 

**Figure 1:**
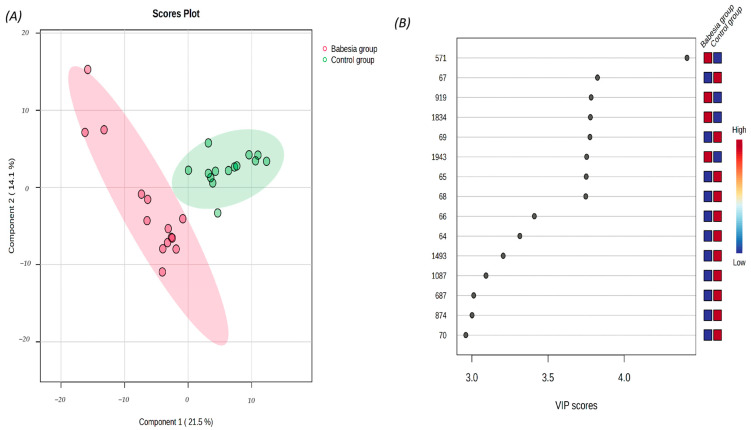
(**A**) The partial least squares discriminant analysis (PLS-DA) score plot of the saliva samples from the control group (green circles) and from the dogs with babesiosis (red circles) in the untargeted LC-MS-based metabolomics approach. (**B**) Variable Importance in Projection (VIP) scores for the 15 most influential features of PLS-DA. The colored boxes on the right represent the relative intensity of each corresponding feature in the respective group. The numbers on the left indicate the peak number, as provided in Supplemental Table S1.

The partial least squares discriminant analysis (PLS-DA) facilitated data visualization based on the presence of infection (Figure 1A), revealing a clear separation between the dogs in the control and the dogs in the babesiosis group. The optimal classification model was built using five components, achieving high performance metrics (R^2^ = 0.99, Q^2^ = 0.69). Notably, a two-component model also demonstrated satisfactory predictive accuracy (R^2^ = 0.85, Q^2^ = 0.68).

The variable importance in projection (VIP) scores highlighted the 15 most influential features driving the discrimination between the healthy and the diseased dogs. Among these, two metabolites, hypoxanthine (peak 64) and inosine (peaks 66 and 1493), were identified as significant contributors to group differentiation (Figure 1B).

**Figure 2:**
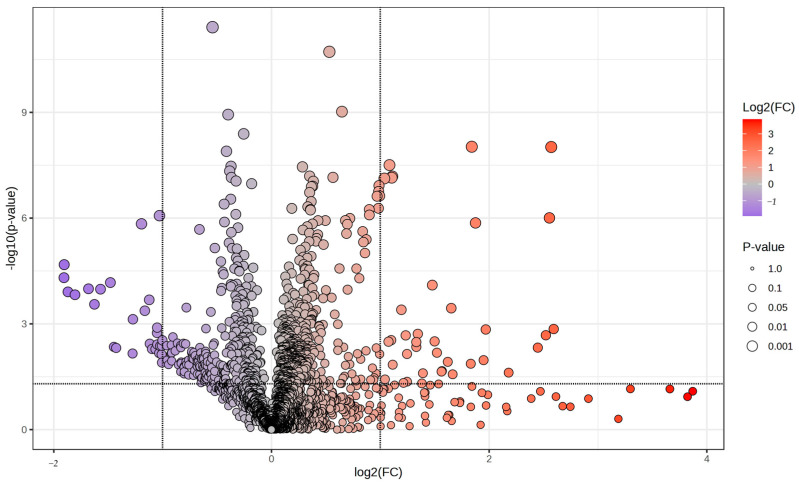
Volcano plot showing features with differential intensities between the control and the babesiosis group. The red points represent features with significantly higher levels in the infected group, while the blue points indicate significantly lower levels. Features that do not meet the significance threshold are shown in gray.

A *t*-test (*p* < 0.05) revealed that 868 features were significantly altered between the groups, while seven metabolites were identified by comparison to reference standards (Table 1). Applying a fold change threshold of 2.0, 37 features showed higher intensity, while 28 exhibited lower intensity, in the babesiosis group compared to the control group, as illustrated in the volcano plot (Figure 2).

### 3.2. Targeted Metabolomics

**Figure 3:**
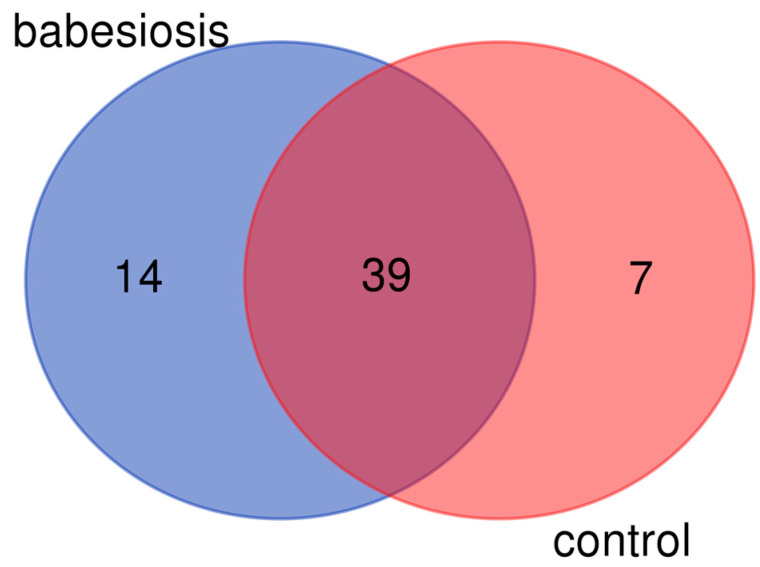
Numbers of valid metabolites identified by targeted metabolomics approach in babesiosis and control group, shown as Venn diagram.

The targeted metabolomics data were processed and analyzed using Biocrates MetIDQ software (version Boron, Biocrates Life Sciences AG, Innsbruck, Austria). Metabolites for which the concentration values were below the LOD in more than 50% of cases in any group were excluded from further statistical analyses, resulting in a reduced dataset of 39 metabolites (Supplementary Table S3). Additionally, 14 metabolites were detected exclusively in the babesiosis group, while seven metabolites were identified and quantified only in the control group (Table 2). The numbers of valid metabolites identified through the targeted metabolomics approach in the babesiosis and the control groups are illustrated using a Venn diagram (Figure 3). This visualization highlights the overlap and unique metabolites detected in each group, emphasizing the metabolic distinctions associated with the disease state.

**Figure 4:**
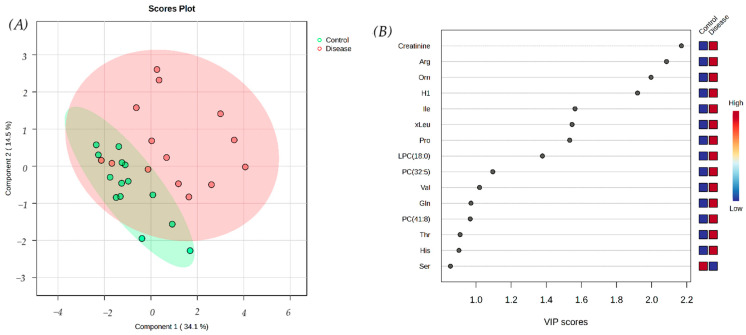
(**A**) The partial least squares discriminant analysis (PLS-DA) score plot of the saliva samples from the control group (green circles) and the dogs with babesiosis (red circles) in the targeted LC-MS-based metabolomics approach. (**B**) Variable Importance in Projection (VIP) scores for the 15 most influential features of PLS-DA. The colored boxes on the right represent the relative concentration of each corresponding metabolite in the respective group.

Univariate and multivariate statistical analyses were applied to the 39 metabolites common to both groups. The PLS-DA plot revealed a partial overlap between the babesiosis and the control groups, indicating some shared metabolic features (Figure 4A). However, the VIP scores identified metabolites with the highest discriminatory power between the groups, such as creatinine, arginine, and ornithine (Figure 4B). These metabolites play a critical role in distinguishing diseased individuals from healthy individuals.

**Figure 5:**
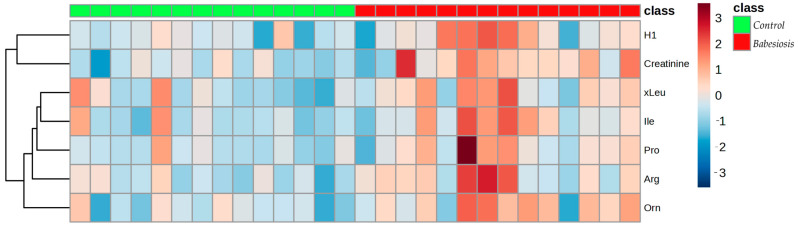
The hierarchical cluster analysis (HCA) based on the metabolites with significantly differential concentrations between the control (green panel) and the babesiosis group (red panel), using Euclidean as a distance measure and Ward as a clustering algorithm. Each colored cell on the map represents the concentration of a specific metabolite, with red indicating higher concentrations and blue indicating lower concentrations.

A *t*-test (*p* < 0.05) revealed that seven metabolites were significantly changed between the groups (Table 3). All the metabolites had higher concentrations in the babesiosis group compared to the control group, as illustrated in the heat map (Figure 5).

The correlation between serum creatinine concentrations and the salivary concentrations determined by targeted metabolomics were calculated by the non-parametric Spearman’s rank correlation coefficient. A very strong positive correlation was found (*p* < 0.05, r = 0.85).

The enrichment analysis of 13 significant metabolites (H1 and xLeu were excluded), identified by untargeted and targeted MS-based metabolomics approaches, showed four different pathways considered to be significant with *p* < 0.05, demonstrating that significant metabolites participated in arginine and proline metabolism, glycine and serine metabolism, the urea cycle, and purine metabolism (Supplemental Table S4).

## 4. Discussion

Host–pathogen interactions are fundamentally driven by the exchange of nutrients between the host cell and the infectious agent. Obligate intracellular pathogens, such as *Babesia*, have undergone coevolution with their hosts, resulting in sophisticated mechanisms to access essential nutrients, evade the host immune response, and establish a protected niche for intracellular replication. Conversely, host organisms have evolved strategies to restrict pathogen replication, often by limiting access to the key nutrients necessary for pathogen survival. Through a comprehensive mass spectrometry-based metabolomics analysis, we demonstrated that *B. canis* infection significantly alters the levels of numerous metabolites in saliva. These metabolites are involved in major metabolic pathways, including amino acid and nucleotide metabolism, energy production, and lipid metabolism. These findings highlight the intricate interplay between the host and the pathogen, encompassing nutrient acquisition and metabolic turnover.

Amino acids are crucial for survival, not only as building blocks of proteins but also as key molecules for the efficient operation of metabolic processes. Recent advances in technology have deepened our understanding of the balance between biosynthesis and the acquisition routes of amino acids in animal-infecting pathogens. In our study, several amino acids showed higher concentrations in the *B. canis*-infected group compared to the control, including arginine, (iso)leucine and proline. Similar to other apicomplexans, the in silico analysis of *Babesia* spp. genomes indicated the absence of de novo amino acid synthesis pathways. However, hemoglobin may serve as a source for the parasite to obtain the necessary amino acids [26,27]. While amino acids are widely recognized for their role in protein production, recent studies have also shed light on the interactions between amino acid metabolism and the regulation of the host’s immune response [28]. 

*Plasmodium* spp. utilize an alternate pathway for the de novo synthesis of proline via arginine degradation. In this pathway, arginine is converted to ornithine by arginase, and ornithine is further processed into proline through ornithine aminotransferase (OAT) and pyrroline-5-carboxylate reductase [29,30]. However, due to the abundance of amino acids from hemoglobin digestion during intraerythrocytic stages, the necessity of this pathway remains unclear. Increased proline concentrations may result from enhanced synthesis or reduced catabolism by proline oxidase and dehydrogenase, which is inhibited by lactate. High lactate levels, documented in canine babesiosis caused by *B. canis*, can increase proline concentration, potentially contributing to albuminuria and glomerular damage [13,31,32].

Arginine is metabolized into ornithine and carbamoyl phosphate through reverse reactions. In protozoa, arginase typically generates ornithine for polyamine biosynthesis [33]. Higher concentrations of ornithine were also detected in the *B. canis*-infected group, suggesting a preference for this pathway. This catabolism may reduce the host’s arginine availability for nitric oxide (NO) synthesis, protecting the parasite from the host’s cytotoxic defences [34]. A comparable mechanism reducing NO production and impairing the deformability of infected erythrocytes has also been observed in *P. falciparum*. In a *P. falciparum* blood-stage developmental cycle metabolomics study, a rapid and specific degradation of arginine mediated by plasmodial arginase was identified [35]. The observed high activity levels of arginase in *Plasmodium* suggest that the parasite depletes the host’s arginine pool to supress the activity of the host’s enzyme, nitric oxide synthase (NOS). Additionally, reduced NO levels may upregulate the host’s endothelial cell receptors, like intercellular adhesion molecule–1 (ICAM-1), promoting the cytoadherence of parasitized red blood cells to the vascular endothelium, further supporting parasite survival and propagation [34]. Similar observations of endothelial activation and the increased expression of cell adhesion molecules, such as ICAM-1, have previously been reported in canine babesiosis [36]. Furthermore, arginine plays a crucial role in activating the hepatic urea cycle for ammonia detoxification, as evidenced by the identification of the urea cycle as one of the most significant metabolite sets in this study.

The de novo synthesis pathway for the branched-chain amino acids leucine, isoleucine, and valine from pyruvate exists in both animals and plants; however, no member of the *Apicomplexa* can synthesize these amino acids [28]. The uptake of isoleucine alongside the concurrent secretion of leucine has been investigated in *P. falciparum* [37]. Isoleucine is the only amino acid that cannot be obtained through hemoglobin degradation during the erythrocytic stage of *Plasmodium* spp. Notably, *P. falciparum* responds to isoleucine starvation by entering a hibernation-like state [26,38,39]. High concentrations of branched-chain amino acids, such as isoleucine and leucine, are also linked to oxidative stress and inflammation in various pathological conditions. Studies have shown that these amino acids can trigger reactive oxygen species (ROS) generation and NF-κB activation in peripheral blood mononuclear cells, as well as inducing inflammation and oxidative stress in endothelial cells, leading to inflammatory cell adhesion and endothelial dysfunction [40]. Nuclear factor kappa-light-chain-enhancer of activated B cells (NF-κB) activation drives the release of pro-inflammatory molecules, such as IL-6, TNF-alpha, ICAM-1, and IL-8, previously associated with babesiosis [36,41,42,43]. Thus, altered isoleucine levels may contribute to the inflammation and oxidative stress observed in babesiosis.

Non-proteinogenic amino acids, such as ornithine and cysteic acid, play essential roles in cellular functions, serving as intermediates in biosynthetic pathways, neurotransmitters, and even toxins [44]. As previously mentioned, arginine and ornithine are precursors for polyamine synthesis. Polyamines are essential for mammalian cell growth and proliferation, playing key roles in DNA replication, RNA transcription, protein synthesis, and post-translational modification. They are linked to biological processes that regulate cellular proliferation, differentiation, and apoptosis [45].

Serum creatinine levels are a key indicator of renal function and are often elevated in canine babesiosis, particularly in cases complicated by acute kidney injury (AKI) [46]. The primary contributors to renal hypoxia and subsequent renal damage in babesiosis are anemia and systemic hypotension [47,48]. Preliminary studies suggest that salivary creatinine concentration may serve as a potential biomarker for the diagnosis of chronic kidney disease (CKD), as salivary creatinine levels have been shown to reflect serum concentrations [49,50]. In our study, we detected the high correlation of serum creatinine concentrations with salivary concentrations, as measured by the targeted LC-MS/MS approach, so we can conclude that salivary creatinine concentrations closely reflect serum concentrations.

The intraerythrocytic growth of *Babesia* necessitates significant energy expenditure due to parasite replication within a brief timeframe. Global and targeted metabolomics studies have emphasized the critical role of energy metabolism in apicomplexan parasites [3,51,52,53]. Similar to *Plasmodium*, *Babesia* predominantly relies on glycolysis for energy production and possesses the complete set of enzymes required for this metabolic pathway [54]. Higher concentrations of hexoses, including glucose, were observed in the babesiosis group, further emphasizing the dominance of glycolysis as the primary pathway for energy production.

Glucose uptake in *P. falciparum*-infected RBCs is markedly elevated, increasing by 75–100-fold compared to uninfected RBCs [55,56]. During the asexual stages, the majority of the glucose taken up by the parasite is converted to lactate, which is subsequently excreted into the host cell environment [57]. The disruption of glucose uptake impairs parasite growth, highlighting the critical role of glycolysis in supporting rapid proliferation [58,59]. In erythrocytic stages, *Plasmodium* prioritizes rapid ATP generation through substrate-level phosphorylation and lactate secretion over the more energy-efficient, but slower, mitochondrial oxidative phosphorylation and complete glucose oxidation [60].

The higher malonate levels found in the babesiosis group competitively inhibit succinate dehydrogenase (SDH), disrupting the tricarboxylic acid (TCA) cycle by causing succinate accumulation and reduced fumarate production. This inhibition impairs the electron transport chain (ETC) via decreased electron transfer through Complex II, leading to diminished ATP generation. Consequently, metabolic pathways shift towards glycolysis to compensate for energy deficits. Additionally, succinate accumulation may contribute to increased ROS production, exacerbating oxidative stress and mitochondrial dysfunction [61]. Previous studies have highlighted the significance of oxidative stress in dogs infected with *B. canis* by measuring antioxidant markers and investigating the relationship between paraoxonase 1 activity and the high-density lipoprotein concentration [62,63,64].

Purine metabolism was emphasized in this study as one of the most significant metabolite sets in the study. Purines and pyrimidines play critical roles in biological processes, including RNA and DNA synthesis, signal transduction, translation, energy metabolism regulation, and serving as structural components of coenzymes [65,66]. Accelerated RNA turnover leads to altered nucleoside levels due to inflammation, metabolic imbalance, or malignancy. The intraerythrocytic proliferation of *Babesia* requires a continuous supply of nucleotides to facilitate DNA replication and the formation of daughter parasites. Studies have demonstrated that *Babesia* can incorporate tritiated hypoxanthine, adenine, and adenosine but not uridine, thymine, or thymidine, indicating a differential regulation of purine and pyrimidine metabolism in *Babesia* spp. [67].

Red blood cells lack mitochondria and the ability to synthesize adenine nucleotides de novo, relying instead on the purine salvage pathway. In a *B. divergens* study, differential nucleotide abundance between uninfected RBCs and infected RBCs was found [3]. Guanine, cytidine, uracil, and their derivatives were significantly enriched in infected RBCs, reflecting the parasite’s high nucleotide demand. Another intermediate in the purine salvage pathway, inosine-5′-phosphate, plays a key role in meeting the purine requirements of *Plasmodium* spp. [35]. The lower levels of inosine and hypoxanthine in this study align more closely with the findings from a serum study of dogs with *B. canis* [12]. In saliva, we found lower levels of adenine, hypoxanthine, inosine, and cytidine, possibly reflecting increased consumption in the serum due to an increased nucleotide demand in support of the growth of intraerythrocytic parasites.

In our study, two amino acids, lysine and phenylalanine, appeared only in the babesiosis group. Higher levels of phenylalanine were also detected in the serum and urine of the dogs with *B. canis,* using untargeted and targeted metabolomics approaches [12,13]. Elevated phenylalanine concentrations have been associated with the development of inflammatory diseases [68]. This phenomenon may result from disruptions in phenylalanine metabolism, specifically through the inhibition of hepatic and renal phenylalanine-4-hydroxylase (PAH) activity, which can be impaired by immune activation and inflammatory processes [69]. Lysine levels have been reported to increase in the serum of mice infected with *P. berghei* and in the urine of individuals with malaria [70,71]. This suggests that lysine dysregulation may be associated with apicomplexan infections. 

Lipid metabolism has key roles in canine babesiosis that are relevant to the disease’s pathogenesis. Targeted metabolomics, performed by applying an FIA-MS analysis, detected a total of 11 significantly different compounds detected only in the babesiosis group and classified into the groups of acylcarnitines, lysophosphatidylcholines, glycerophospholipids, triglycerides, and sphingolipids. Additionally, six lipid compounds were detected only in the control group. Similar to other *Apicomplexan*, despite a high demand for lipids, *Babesia* does not synthesize most of its own lipids [54]. 

Membrane lipids play a crucial role in modulating RBC rheology, maintaining membrane deformability and fluidity as RBCs traverse the host’s capillaries. During parasite proliferation within RBCs, there is a substantial demand for lipids to construct the membranes of daughter cells. Recent studies observed significant changes across all lipid classes [3,72,73]. *Babesia bovis*-infected RBCs show increases in phosphatidylcholine, phosphatidic acid, diacylglycerol, and cholesteryl esters compared with uninfected RBCs [73]. During the sexual development of *P. falciparum*, significant alterations occur in the lipid composition of parasite-infected RBCs, enabling the distinction of individual parasite stages based on their unique lipid profiles [72]. Similarly, in *Babesia divergens*-infected RBCs, diverse metabolic pathways involving a wide range of lipid classes have been identified [3]. Lipid profile changes were also detected in the serum and urine of dogs with *B. canis* [12,13].

## 5. Conclusions

Alterations in the blood metabolome can influence the composition of salivary secretions; however, as our findings indicate, these changes are not fully reflected in saliva. Consequently, it was crucial to evaluate the salivary metabolome in canine babesiosis and determine the extent to which saliva serves as an effective indicator of infection. Our findings revealed that canine babesiosis is associated with significant changes in salivary metabolites, reflecting disruptions in amino acid, nucleotide, and lipid metabolism, as well as energy production pathways. Key biochemical processes, such as the urea cycle and purine metabolism, were also impacted. These insights into the metabolic shifts underlying the disease are critical for guiding the development of advanced diagnostic tools and therapeutic strategies in future research.

## Figures and Tables

**Table 1:** A list of the accurately identified and significantly changed metabolites in the saliva of dogs with *B. canis* versus the control group, obtained using the untargeted LC-MS metabolomics approach.

Peak ID	FrAnK Annotation	Mass	RT	Polarity	log2(FC)	*p* Value
25	Adenine	136.0619	502.68	positive	−0.79	0.012
64	Hypoxanthine	137.0459	556.11	positive	−1.63	0.000
66	Inosine	269.0881	556.45	positive	−1.68	0.000
686	Cytidine	244.0928	599.26	positive	−1.05	0.002
1493	Inosine	267.0738	555.21	negative	−1.57	0.000
1998	Cysteic acid	167.9975	736.79	negative	−1.16	0.000
6	Betaine	118.0862	559	positive	−0.42	0.048
1378	Malonate	103.0039	727.34	negative	1.25	0.044

RT—retention time. Log2(FC)—log-ratio of a feature’s intensity values between the babesiosis and the control group.

**Table 2:** The metabolites detected and quantified only in the babesiosis or the control group by targeted metabolomics assessing the saliva of control dogs and dogs with babesiosis. Concentrations are given as means and standard deviations.

Metabolite	Babesiosis Group		Control Group	
(μmol dm^−^³)	Mean	SD	Mean	SD
AC (0:0)	24.19	13.86	NA	NA
AC (4:0)	NA	NA	0.61	0.28
alpha-AAA	13.28	28.36	NA	NA
Cer (34:1)	NA	NA	0.07	0.06
Cer (42:1)	2.69	1.65	NA	NA
LPC (17:0)	2.78	1.76	NA	NA
LPC (20:3)	0.33	0.32	NA	NA
Lys	95.08	159.55	NA	NA
PC (25:0)	NA	NA	0.05	0.02
PC (31:2)	0.31	0.23	NA	NA
PC (32:6)	NA	NA	0.28	0.14
PC (33:2)	1.18	0.41	NA	NA
PC (37:5)	NA	NA	0.28	0.09
PC (40:8)	1.40	1.36	NA	NA
PC-O (34:4)	0.45	0.27	NA	NA
Phe	41.81	58.23	NA	NA
SM (32:2)	NA	NA	0.32	0.19
SM (38:1)	4.30	3.79	NA	NA
t4-OH-Pro	NA	NA	5.46	4.94
TG (51:1)	0.65	0.23	NA	NA
TG (52:7)	1.77	0.79	NA	NA

AC—Acylcarnitines. Alfa-AAA—Aminoadipic acid. Cer—Ceramides. LPC—Glycerophospholipids. Lys—Lysine. PC—Phosphatidylcholine. Phe—Phenylalanine. SM— Sphingomyelins. TG—Triglycerides. NA—not available.

**Table 3:** A list of the significantly changed metabolites in the saliva of dogs infected with *B. canis* compared to the control group, acquired using the targeted LC-MS metabolomics approach.

Metabolite Name	*p* Value	log2(FC)
Creatinine	0.002	1.63
Ornithine	0.005	1.38
Arginine	0.008	2.09
Isoleucine	0.025	0.99
H1 (hexoses, including glucose)	0.035	2.26
Proline	0.038	1.66
xLeu (leucine + isoleucine)	0.049	0.92

Log2(FC)—the log-ratio of the metabolites’ concentrations values between the babesiosis and the control group.

## Data Availability

The original contributions presented in the study are included in the article/Supplementary Materials, further inquiries can be directed to the corresponding author.

## Supplementary Materials

The following supporting information can be downloaded at: www.mdpi.com/article/10.3390/biom15040520/s1, Supplemental Table S1. Peak intensity table with mass, retention time and annotations of features identified by untargeted metabolomics approach in the saliva of control dogs and dogs with *B. canis*. Supplemental Table S2. List of all identified features on the basis of the mass/retention time matched to known standards in the PiMP software in untargeted metabolomics approach. Supplemental Table S3. Concentrations (μmol dm^−3^) of all valid metabolites obtained by targeted metabolomics (AbsoluteIDQ p400 HR Kit, Biocrates LifeSciences AG, Innsbruck, Austria) in the saliva of control dogs and dogs with babesiosis. Supplemental Table S4. Metabolite Set Enrichment Analysis (MSEA) of the significant metabolites identified by the untargeted and targeted approach, examined with the pathway associated metabolite sets (SMPDB; 99 metabolite sets based on normal human metabolic pathways set as metabolite set library).
